# The relationship between perceived social support and childbearing desire among married university students in Southeastern Iran

**DOI:** 10.1186/s12889-026-26947-8

**Published:** 2026-05-11

**Authors:** Parniya Abolghaseminejad, Reza Sadeghi, Vahidreza Borhaninejad, Batool Zeidabadi, Mohammad Moqaddasi Amiri

**Affiliations:** 1 Department of Public Health, Sirjan School of Medical Sciences, Sirjan, Iran; 2 Department of Health Education and Promotion, School of Public Health, Tehran University of Medical Sciences, Tehran, Iran; 3 Social Determinants of Health Research Center, Institute for Futures Studies in Health, Kerman University of Medical Sciences, Kerman, Iran; 4 Sirjan School of Medical Sciences, Sirjan, Iran

**Keywords:** Social Support, Childbearing, Population Growth, Pregnancy, Youthful Population, Iran

## Abstract

**Background:**

Declining fertility rates and changes in family structures have made childbearing desire an important population health concern. Social support, particularly perceived social support, is considered a key factor influencing individuals’ emotional well-being and reproductive intentions. This study aimed to examine the relationship between perceived social support and childbearing desire among married university students in Sirjan, Iran.

**Methods:**

This cross-sectional study was conducted in 2024 among 287 married university students selected through stratified sampling with proportional allocation. Data were collected using a demographic questionnaire, the Childbearing Desire Questionnaire (Cronbach’s alpha = 0.83), and the Medical Outcomes Study–Social Support Survey (MOS-SSS; Cronbach’s alpha range = 0.74–0.93). Data were analyzed using descriptive statistics, Spearman correlation, Mann–Whitney U test, and Kruskal–Wallis test due to non-normal data distribution.

**Results:**

The results showed a significant positive correlation between childbearing desire and perceived social support (*r* = 0.188, *p* = 0.001). Among social support dimensions, emotional/informational support demonstrated the strongest association with childbearing desire (*r* = 0.255, *p* < 0.001). Childbearing desire was also positively associated with age and number of children, while participants with better economic status reported significantly higher childbearing desire (*p* < 0.001).

**Conclusion:**

Perceived social support, particularly emotional and informational support, plays an important role in shaping childbearing desire among married university students. Strengthening social support systems and improving economic conditions may help promote positive reproductive intentions. Married students who feel more supported by family and others are more likely to want children. Policies that improve emotional support and financial stability may encourage childbearing among young couples.

## Introduction

One of the significant concerns of today’s society is the decline in population growth, accompanied by an increase in the middle-aged and elderly population. Over the past half-century, considerable changes have occurred in the structure and characteristics of families, partly due to the transition from extended families to nuclear families, which has consequently led to a decline in birth rates [1]. As a result, childbearing desire has increasingly been recognized as a critical area of population health. This domain can help us understand global family-related issues, such as unintended pregnancies, low childbearing rates, and delays in childbearing [2].

Childbearing is influenced by various factors, including governmental policies and individual, social, economic, and cultural elements [3]. Numerous studies have shown that childbearing desire is associated with the family’s socioeconomic status, increased urbanization, age, education level, and employment status of the couple [1, 2, 4, 5].

Social support is also an important determinant in reproductive decision-making. It can reduce the caregiving burden for children and, in some cases, alleviate financial and psychological pressures on parents [6]. It has two main dimensions: received social support and perceived social support. Received social support refers to the actual support an individual gets, while perceived social support is the individual’s subjective evaluation of the availability of support in times of need. Social support is a qualitative, subjective, and measurable concept. It is influenced by culture, evolution, personality, and support systems [7].

Beyond its definition, several theoretical perspectives explain how social support affects fertility intentions. For example, the Theory of Planned Behavior emphasizes that social norms and perceived support from significant others influence individuals’ intentions to have children [8]. Similarly, the Social Capital Theory suggests that social networks and trust within communities can encourage family formation by providing emotional and instrumental resources [9]. These frameworks highlight that perceived social support not only provides practical assistance but also reinforces confidence and readiness for parenthood [10].

Perceived social support plays a significant role in individuals’ psychological well-being. However, previous studies present conflicting evidence, including the influence of cultural differences on social support patterns [7]. On the other hand, childbearing remains one of the critical challenges in population studies and is a highly significant issue in social matters [1]. It necessitates examination in light of influencing factors such as social, demographic, and economic characteristics [3]. In Iran, the trend of declining fertility has drawn considerable policy and research attention. However, the extent to which perceived social support contributes to the childbearing intentions of young adults is still underexplored [11]. Therefore, to address this gap, the present study aimed to determine the relationship between perceived social support and childbearing desire among married university students in Sirjan County in 2024.

## Methods

This cross-sectional survey was conducted among married university students in Sirjan County, Kerman Province, Iran, from mid-October 2024 to early December 2024.

### Sample

The study population consisted of all married students enrolled at the School of Medical Sciences, Islamic Azad University, Payame Noor University, and University of Technology in Sirjan County. A census approach was applied, and all eligible married students were invited to participate in the study.

Although a minimum sample size of 270 participants was estimated based on previous studies, a census approach was ultimately adopted to include all eligible married students.

### Data collection

Data were collected from all eligible married students enrolled at universities in Sirjan County using a census approach. Initially, 300 married students were invited to participate in the study. After excluding 13 questionnaires due to incomplete responses, data from 287 participants were included in the final analysis.

Data were collected using a demographic information form, the Childbearing Desire Questionnaire, and the Medical Outcomes Study–Social Support Survey (MOS-SSS). The demographic form included questions on age, gender, duration of marriage, educational level, occupation, economic status, number of children, history of miscarriage, history of unintended pregnancy, spouse’s education, and spouse’s occupation.

The Childbearing Desire Questionnaire, developed by Enayat in 2013 [12], consists of 27 items rated on a five-point Likert scale, with total scores ranging from 27 to 135. Higher scores indicate greater childbearing desire. The validity and reliability of this questionnaire have been confirmed in previous studies, with a reported Cronbach’s alpha of 0.83 [13].

Perceived social support was assessed using the Medical Outcomes Study–Social Support Survey (MOS-SSS), developed by Sherbourne and Stewart. This instrument includes 19 items covering emotional/informational support, tangible support, positive social interaction, and affection. Items are rated on a five-point Likert scale, with total scores ranging from 19 to 95. Previous studies have reported acceptable reliability for this instrument, with Cronbach’s alpha values ranging from 0.74 to 0.93 [14].

After obtaining ethical approval from the Ethics Committee of Sirjan School of Medical Sciences (IR.SIRUMS.REC.1402.025) and receiving official permissions from the relevant university authorities, a list of married students was obtained from university administrative offices. Several trained interviewers administered paper-based questionnaires in person at the respective universities. Written informed consent was obtained from all participants prior to data collection. Participants were assured of anonymity and confidentiality, and each questionnaire took approximately 15 min to complete.

### Data analysis

For data analysis, descriptive statistics, including mean and standard deviation, were used. Due to the data’s non-normality, the Spearman correlation coefficient, Mann-Whitney, and Kruskal-Wallis tests were applied using SPSS version 27 for inferential analysis. A significance level of less than 0.05 was considered for all tests.

## Results

The demographic characteristics of the participants are presented in Table 1. Overall, most participants were female, enrolled in undergraduate programs, and studying at different universities in Sirjan County. The majority reported an average economic status, and self-employment was the most common occupational category. The mean age and duration of marriage indicated that participants were predominantly young adults in the early years of marriage.

**Table 1:** Description of demographic variables of married student participants (*n* = 287)

Variable	Category	Frequency	Percentage
Gender	Female	182	63.4
	Male	105	36.6
Education Level	Associate Degree	46	16.04
	Bachelor’s Degree	214	74.56
	Master’s Degree	27	9.40
University	Islamic Azad University	78	27.2
	Payam Noor University	70	24.4
	University of Technology	74	25.8
	School of Medical Sciences	65	22.6
Occupation	Unemployed	98	34.1
	Government Employee	71	24.7
	Self-employed	118	41.2
Economic Status	Good	60	20.9
	Average	177	61.7
	Poor	50	17.4
History of Miscarriage	Yes	59	20.6
	No	228	79.4
History of Unintended Pregnancy	Yes	32	11.1
	No	255	88.9
Spouse’s Education	Below Diploma	14	4.9
	Diploma	26	9.1
	Associate	59	20.5
	Bachelor’s	156	54.4
	Above Bachelor’s	32	11.1
Spouse’s Occupation	Unemployed	45	15.7
	Government Employee	89	31.0
	Self-employed	153	53.3

The Kolmogorov–Smirnov test indicated that childbearing desire and perceived social support were not normally distributed (Table 2). Therefore, non-parametric statistical tests were used for subsequent analyses.

**Table 2:** Kolmogorov-Smirnov test for normality of dependent variables (*n* = 287)

Variable	Kolmogorov-Smirnov Statistic	df	*p*-value
Childbearing Desire	0.148	287	< 0.001
Social Support	0.132	287	< 0.001

Spearman correlation analysis revealed a significant positive association between overall perceived social support and childbearing desire. Among the dimensions of social support, only emotional and informational support showed a significant relationship with childbearing desire, whereas other dimensions were not significantly associated (Table 3).

**Table 3:** Spearman correlation between childbearing desire and social support (*n* = 287)

Variable	Spearman’s *r*	*p*-value
Total Social Support	0.188	0.001
Emotional/Informational Support	0.255	< 0.001
Tangible Support	0.042	0.48
Positive Social Interaction	0.037	0.54
Affection	0.015	0.81

The mean scores of childbearing desire and perceived social support, along with their respective dimensions, are summarized in Table 4.

**Table 4:** Mean scores of childbearing desire and social support (*n* = 287)

Variable	Mean	Standard Deviation	Observed Range		Expected Range	
			Maximum	Minimum	Maximum	Minimum
Childbearing desire	85.69	9.23	127	42	135	27
Social support	63.01	9.86	95	27	95	19
Emotional/Informational Support	26.13	4.56	40	8	40	8
Tangible Support	14.04	2.67	20	4	20	4
Positive Social Interaction	9.96	2.05	15	3	15	3
Affection	9.69	1.83	15	4	15	3

The relationships between childbearing desire and demographic variables are presented in Table 5. No significant differences in childbearing desire were observed according to gender, educational level, university, occupation, history of miscarriage, history of unintended pregnancy, or spouses’ educational level and occupation. In contrast, childbearing desire differed significantly according to participants’ self-reported economic status, with higher levels observed among those reporting a good economic status.

**Table 5:** Relationship between fertility desire and demographic variables (*n* = 287)

Variable	Category	Frequency	Mean ± SD	Test Statistic	*p*-value
Gender	Female	182	86.06 **±** 9.67	-0.891	0.37*
	Male	105	85.05 **±** 8.43		
History of miscarriage	Yes	59	87.01 **±** 7.93	-1.041	0.29*
	No	228	85.35 **±** 9.53		
History of Unintended Pregnancy	Yes	32	86.68 **±** 11.90	-1.405	0.16*
	No	255	85.57 **±** 8.87		
Education level	Associate	46	87.06 **±** 11.74	0.313	0.855*
	Bachelor’s	214	85.43 **±** 8.76		
	Master’s	27	85.44 **±** 8.21		
University	Islamic Azad University	78	86.12 **±** 11.03	4.318	0.22**
	Payam Noor University	70	84.78 **±** 10.15		
	University of Technology	74	86.94 **±** 8.72		
	School of Medical Sciences	65	84.73 **±** 5.75		
Occupation	Unemployed	98	84.88 **±** 7.78	2.427	0.29**
	Government Employee	71	85.94 **±** 7.90		
	Self-employed	118	86.22 **±** 10.31		
Economic Status	Good	60	90.28 **±** 9.11	18.318	< 0.001**
	Average	177	84.70 **±** 9.54		
	Poor	50	83.70 **±** 6.12		
Spouse’s educational level	Below Diploma	14	85.57 **±** 6.06	4.142	0.387**
	Diploma	26	82.46 **±** 8.68		
	Associate	59	86.27 **±** 8.10		
	Bachelor’s	156	86.08 **±** 9.82		
	Above Bachelor’s	32	85.43 **±** 9.77		
Spouse’s Occupation	Unemployed	45	86.06 **±** 8.06	0.960	0.619**
	Government Employee	89	86.02 **±** 8.67		
	Self-employed	153	85.39 **±** 9.89		

* *p*-value of Mann-Whitney Test

** *p*-value of Kruskal-Wallis Test

## Discussion

To maintain a young and dynamic population, it is essential to identify the factors related to this area, especially among individuals of childbearing age [3]. The present study also investigated childbearing desire among married universities students in Sirjan County and examined its relationship with perceived social support.

The results of the present study showed that childbearing desire among the participants was at a moderate level. In line with this finding, studies conducted by Karimian and Hejazi in Zanjan and Faraji et al. in Ilam reported that the participants’ childbearing desire was also moderate [15, 16]. Additionally, a study on women residing in the cities of Nowshahr and Chalus showed that only 47% of mothers desired another child [17], which aligns with the present study. However, other studies reported results contrary to the present study, indicating a lower childbearing desire. For instance, in a study by Zhu et al., the intention to have a second or third child among pregnant couples in Shanghai, China, was reported to be as low as 16.2% and 9.4%, respectively [3]. The difference between this study and the present one may be attributed to variations in research tools, target populations, and cultural differences. People in different cities and countries decide to have children based on their attitudes and preferences, and social and economic factors play a crucial role in the level of childbearing desire.

Additionally, the results of the present study indicated that the level of perceived social support was moderate. In the study by Azimi et al., the level of perceived support in pregnant women was reported as 77.90%, also indicating a moderate level [7]. However, the study by Ali Dosti et al. in Kermanshah revealed that 18.9% of respondents reported low levels of social support, 43.9% reported moderate levels, and 37.7% reported high levels of social support [18]. The differences in sample size and the tools used may have contributed to these discrepancies. Generally, social support is influenced by living conditions, socioeconomic status, cultural differences, and ethnic and racial dependencies. It can come from various sources, such as family, friends, neighbors, and colleagues.

Other results indicated a positive and significant relationship between childbearing desire and social support, meaning that the higher the perceived social support in the participants, the greater their desire to have children. Additionally, the analysis of the relationship between childbearing desire and the dimensions of social support showed that childbearing desire was only positively and significantly related to emotional support/informational support. The study by Ali Dosti et al. also found that social support was the only variable that positively impacted childbearing desire [18]. Similarly, in the survey by Abbasi Shavazi et al., it was found that some supportive variables, such as receiving help with household chores from the spouse, receiving help with household chores from the husband’s parents, and the frequency of in-person contact with the husband’s parents, had an impact on increasing women’s childbearing desire [17]. These findings align with the results of the current study.

The sense of connection with the family, parental support and supervision, and positive parent-child relationships lead to greater childbearing desire among young people [1]. For example, married students who rely on the support of their parents are more likely to have a childbearing desire because they have a reliable support system [6]. Despite the spread of modernization and the shift from extended families to nuclear families, Iranian families have always maintained their supportive role toward their children. With increasing economic difficulties, families struggle to fulfill their intergenerational supportive role toward their children [19].

The results showed a positive and significant relationship between childbearing desire and age, as well as the number of children; that is, the higher the age of the participants or the more children they had, the greater their desire for more children. In the study by Rahman et al., the age of the women participants played a role in their childbearing desire [4]. Similarly, in the study by Zhu et al., age was identified as one of the most critical factors associated with having a second child. Still, their findings showed that women aged 35 and older were less likely to desire a second child [3]. The study by Abbasi Shavazi et al. also found that mothers with one child were more likely to want another child [17]. These findings are consistent with the results of the present study.

In this study, it was found that there was no significant difference in childbearing desire across different educational levels, universities, or employment types. Additionally, no significant difference was found between childbearing desire and participants’ spouses’ educational levels or employment status. However, in the study by Rahman et al., variables such as education, occupation, income, and ethnicity simultaneously played a role in women’s childbearing desire [4]. In the study by Brauner-Otto et al., conducted among young people aged 18 to 27 years, it was observed that individuals with lower levels of education were uncertain about whether they would have children. On the other hand, among those who anticipated having children, those with higher levels of education intended to delay childbearing [2]. Similarly, Abbasi Shavazi et al. found that women with lower levels of education (e.g., below secondary school) had higher childbearing desires than those with higher educational levels (diploma, associate, and bachelor’s) [17]. To explain these results, it can be said that education exposes individuals to different choices and significantly impacts women in society. Higher education and advanced degrees are often associated with fewer births and longer intervals between births [4]. The discrepancy between these findings and the present study may be attributed to differences in the study populations, cultural and social factors, and different research tools. Additionally, most of the studies reviewed used researcher-designed questionnaires to assess childbearing desire, which may account for some of the differences in the results.

From the participants’ perspectives, this study showed a significant difference between childbearing desire and economic status levels. Specifically, those who evaluated their economic status as good had the highest childbearing desire, while those who reported their economic status as weak had the lowest childbearing desire. In the study by Smith et al., Nigerian men cited economic pressure and changes in expectations as the main reasons for delaying marriage and childbearing. They identified money as essential for successful reproduction [5]. In the study by Brauner-Otto et al., it was found that both men and women with lower incomes and greater concerns about their future employment were uncertain about their decision to have children [2]. Zhu et al. also found that financial limitations hurt the intention to have a third child [3].

Economic conditions or underlying factors such as employment status, income, and job security have long been associated with childbearing behaviors. Recent evidence indicates a significant and bidirectional relationship between economic conditions and childbearing outcomes. Today’s youth face greater economic instability and reduced job security [2]. However, financial stability is undoubtedly essential for raising children. It is also crucial for couples to be aware that delaying childbearing may place them in even greater financial challenges for reproduction [20]. Policymakers and planners must address the economic barriers to childbearing and consider strategies to influence individuals’ early decisions and intentions regarding childbearing [2]. Furthermore, as socio-economic development positively impacts reproductive health during childbirth [4], providing incentives such as special childbearing insurance, financial support, and social assistance, like maternity leave, could encourage childbearing [3].

### Limitations

Like other studies conducted in the field, this study had some limitations. For example, this research was conducted only at universities in Sirjan, so to improve the generalizability of the results, it is recommended that future studies investigate childbearing intentions among married students at universities across different provinces in the country and compare the findings with each other. Additionally, given that factors such as the cost of children’s education [3], social media [6], and ethnicity [4] have been shown to influence childbearing intentions in previous studies, it is suggested that these factors be included in future research as well.

## Conclusions

The results of the present study showed that childbearing intentions were higher among individuals with stronger social support, older age, and a greater number of children. Additionally, individuals with higher economic status had a greater desire for childbearing. Accordingly, policymakers and planners in population youth policies must pay special attention to these two influential variables. Practical steps should be taken to strengthen perceived social support and improve the economic conditions of married students. It is hoped that the findings of this study will serve as a reference or guide for predicting childbearing and childbearing intentions among married students at universities across the country.

## Data Availability

All data generated or analysed during this study are included in this published article.
